# *emm* Types and clusters and macrolide resistance of pediatric group A streptococcal isolates in Central Greece during 2011-2017

**DOI:** 10.1371/journal.pone.0232777

**Published:** 2020-05-07

**Authors:** Ioanna N. Grivea, George A. Syrogiannopoulos, Aspasia N. Michoula, Georgia Gazeti, Ergina Malli, Katerina Tsilipounidaki, Sotirios Fouzas, Michael B. Anthracopoulos, Efthymia Petinaki

**Affiliations:** 1 Department of Pediatrics, Faculty of Medicine, School of Health Sciences, University of Thessaly, Biopolis, Larissa, Greece; 2 Department of Microbiology, Faculty of Medicine, School of Health Sciences, University of Thessaly, Biopolis, Larissa, Greece; 3 Department of Pediatrics, Faculty of Medicine, School of Health Sciences, University of Patras, Rion-Patras, Greece; Universidade de Lisboa Faculdade de Medicina, PORTUGAL

## Abstract

**Background:**

The surveillance of *emm* types and macrolide susceptibility of group A streptococcus (GAS) in various areas and time periods enhances the understanding of the epidemiology of GAS infections and may guide treatment strategies and the formulation of type-specific vaccines. Greece has emerged as a country with high macrolide use. However, studies suggest a gradual reduction in macrolide consumption after 2007.

**Methods:**

During a 7-year period (2011–2017), 604 GAS isolates were recovered from consecutive children presenting with pharyngeal or nonpharyngeal infections in Central Greece; 517 viable isolates underwent molecular analysis, including *emm* typing.

**Results:**

Isolates belonged to 20 different *emm* types (in decreasing order of prevalence: 1, 89, 4, 12, 28, 3, 75 and 6, accounting for 88.2% of total isolates). The *emm* types comprised 10 *emm* clusters (five most common clusters: E4, A-C3, E1, A-C4 and A-C5). The *emm*89 isolates were acapsular (‘new clade‘). Overall macrolide resistance rate was 15.4%, and cMLS_B_ emerged as the predominant resistance phenotype (56.4%). The lowest annual resistance rates occurred in 2014 (13.1%), 2016 (5.5%) and 2017(8.0%) (*P* for trend = 0.002). Consumption of macrolide/lincosamide/streptogramin B declined by 22.6% during 2011–2017. Macrolide resistance and *emm*28 and *emm*77 types were associated (both *P*<0.001). The most frequently identified genetic lineages of macrolide-resistant GAS included *emm*28/ST52, *emm*77/ST63, *emm*12/ST36, *emm*89/ST101 and *emm*4/ST39. We estimated that 98.8% of the isolates belonged to *emm* types incorporated into a novel 30-valent M protein vaccine.

**Conclusions:**

In Central Greece during 2011–2017, the acapsular *emm*89 isolates comprised the second most prevalent type. Susceptibility testing and molecular analyses revealed decreasing GAS macrolide resistance rates, which may be attributed to the reduction in the consumption of macrolides and/or the reduced circulation of macrolide-resistant clones in recent years. Such data may provide valuable baseline information in targeting therapeutic intervention and the formulation of type-specific GAS vaccines.

## Introduction

*Streptococcus pyogenes* [group A streptococcus (GAS)] infections are a major cause of morbidity and mortality worldwide and are responsible for a diverse array of noninvasive, invasive and immune-related diseases [1]. The M protein of GAS is a surface protein, which constitutes a major virulence factor as well as the major immunologic epitope of GAS [2]; it possesses a hypervariable region of the amino-terminal, 40 to 50 amino acid residues. The gene encoding M protein, *emm*, is the basis for sequence typing used to differentiate among strains of GAS; this differentiation is founded on relatively minor sequence differences in the 5ʹ regions of the gene [3–6]. More than 220 *emm* types have been recognized to date [7].

A protein-based multivalent type-specific GAS vaccine containing amino-terminal M peptides from 30 different *emm* types has been developed [8, 9]. N-terminal M protein peptides evoke protective antibodies against epidemiologically important GAS types with the greatest bactericidal activity.

Recently, an *emm* cluster system based on strong phylogenetic support has been described, which serves as a functional classification scheme for GAS M proteins [10]. It adds valuable information regarding tissue tropism and immune response to GAS infections in various settings on a worldwide level. The *emm* cluster system is expected to guide vaccine design when attempting to predict vaccine efficacy [10–12]. This system has already been used to analyze the GAS epidemiology in the Pacific region, which is characterized by a great variety of circulating *emm* types [11].

The most important known mechanisms implicated in GAS macrolide resistance are a 14- and 15-membered ring macrolide-specific efflux (M phenotype) [13], encoded by the *mef*(A) gene [14], as well as the modification of the ribosomal target by a methylase encoded by the *erm*(B) [15] or the *erm*(TR) gene [16]; the latter is currently referred to as *erm*(A) or *erm*(A) subclass *erm*(TR) gene [17]. Methylation results in reduced binding of and co-resistance to 14-, 15-, and 16-membered ring macrolide, lincosamide, and streptogramin B (MLS_B_) antibiotics. Methylase can be expressed either constitutively (cMLS_B_ phenotype) or be induced (iMLS_B_ phenotype).

In several European countries increased rates of macrolide-resistant GAS isolates have been recorded in the late 1990s and early 2000s [18–24]. In these countries various dynamics have been observed −from 2005 onwards− regarding the prevalence of macrolide resistance, the phenotypes involved, the *emm* type distribution and the circulating resistant clones [25–28]. Therefore, continuing surveillance is in order as it will enhance the understanding of the epidemiology of GAS disease and may guide the formulation of multivalent type-specific vaccines.

High rates of macrolide-resistant GAS isolates have repeatedly been reported in Greece as compared to other European countries [29, 30]. The University General Hospital of Larissa (UGHL) serves as the academic tertiary care referral center for the broader area of Central Greece. The aim of this 7-year survey was to investigate three clinically important epidemiological characteristics of pharyngeal and nonpharyngeal GAS isolates: (i) *emm* type and cluster distribution, (ii) phenotypes, genotypes and clones of macrolide-resistant isolates, and (iii) proportion of isolates covered by the currently proposed 30-valent M protein-based GAS vaccine.

## Materials and methods

### Ethics statement

The research protocol was approved by the Ethics Committee of UGHL. The data was analyzed anonymously.

### Patients and specimens

During the period January 1, 2011 to December 31, 2017, 604 GAS isolates were recovered from consecutive pediatric patients, who presented with pharyngeal or nonpharyngeal infections to the outpatient pediatric clinic and/or were hospitalized in the pediatric ward of UGHL. The population pool was derived from regions of Central Greece. In UGHL, it has been routine practice to perform swab culture on all children presenting with signs and symptoms of streptococcal pharyngitis. A single GAS isolate was identified in each child; each isolate was derived from a single pharyngeal or nonpharyngeal location.

### Antimicrobial susceptibility testing and detection of macrolide resistance genes

Isolates were identified as GAS by colony morphology, β-hemolysis on sheep blood agar and Lancefield grouping with the use of a commercially available agglutination technique (Slidex, Streptokit; BioMérieux, Marcy l' Etoile, France). Susceptibility testing of isolates to benzylpenicillin, erythromycin and clindamycin was performed according to EUCAST guidelines (www.eucast.breakpoints v10.0). D test was performed in order to differentiate the iMLS_B_ phenotype from the M phenotype. The D test was performed by disk diffusion, i.e. by placing a 15 μg erythromycin disk in 12–16 mm proximity to a 2 μg clindamycin disk on a Mueller Hinton agar plate which was inoculated with a streptococcal isolate; detection of the iMLS_B_ phenotype was indicated by blunting of the clindamycin inhibition zone (www.eucast.breakpoints v10.0).

Streptococcal DNA was extracted according to CDC laboratory protocols for *S*. *pyogenes* (http://www.cdc.gov/streplab/protocols.html). All viable erythromycin-resistant isolates were screened for the presence of genes associated with macrolide resistance by Polymerase Chain Reaction (PCR), using specific primers for *erm*(B), *erm*(TR) and *mef*(A) [31, 32]. PCR products were separated in 2% gel electrophoresis for 30–40 minutes and samples were identified as positive if they exhibited a clearly defined band at 639bp, 371bp and 348bp, respectively.

### Molecular typing

A total of 517 isolates were initially classified by *emm* typing [6]. The *emm* types were determined by amplification of the M protein gene (*emm*). All protocols and assignment of *emm* types and subtypes were as described in the *S*. *pyogenes emm* sequence database (http://www.cdc.gov/streplab/protocol-emm-type.html). The assignment of *emm* clusters was based on the CDC database (http://www.cdc.gov/streplab/downloads/distribution-emm-types.pdf). The isolates which belonged to type *emm*89 were further characterized for the presence of the *hasABC* locus, which is responsible for the synthesis of the hyaluronic acid capsule, using two different pairs of primers. In ‘new clade’ *emm*89 isolates, which lack the presence of *hasABC* locus, the first primer pair amplified across the *hasABC* locus from surrounding genes and generated a product of 632bp; the second primer pair amplified within a 157bp region, which is present only in clade-associated *emm*89 isolates −in place of the *hasABC* locus− and generated a product of 127bp [33].

All macrolide-resistant isolates and seven randomly selected macrolide-susceptible ones which belonged to *emm*89 −each study year represented by one isolate− were, in addition, characterized by Multilocus Sequence Typing (MLST) of the seven gene loci *gki*, *gtr*, *murI*, *mutS*, *recP*, *xpt* and *yqiL* [34]. The data for *S*. *pyogenes* alleles and sequence types (STs) was obtained through the MLST database at https://pubmlst.org/spyogenes/. Electrophoresis, purification and sequencing of PCR products for *emm* typing and MLST were performed as described above. New *emm* subtypes and new MLST alleles and STs were assigned to https://www2.cdc.gov/vaccines/biotech/strepblast.asp and https://pubmlst.org/spyogenes/, respectively.

### Consumption of macrolides

The IMS Health Xponent Greece database was used to obtain data on the annual outpatient consumption of total and pediatric suspensions packages of macrolide, lincosamide and streptogramin B, expressed in packages per 1,000 inhabitants per day [35].

### Statistical analysis

For comparison between groups categorical parameters were compared using the Fisher’s exact test. We assessed the difference between macrolide-susceptible and macrolide-resistant rates for each *emm* type using the standardized residuals *post hoc* method for contingency tables [36], with Bonferroni adjustment for multiple comparisons (critical *P* value = 0.0025). Yearly fluctuation was tested using a log-linear model, in which the exploratory variable was year effect and the response was measured as frequency of a certain *emm* type or rate of macrolide-resistant isolates. Difference in trend over time was analyzed by the χ^2^ test for trend, and the association between parameters was analyzed by Pearson’s correlation test. Two-sided tests were used. All analyses were performed with the IBM SPSS software version 25.0 (IBM Corp., Armonk, NY). An effect was considered significant when *P*<0.05.

## Results

### Sample and patient characteristics

During the 7-year study period, 604 GAS isolates were obtained from children with pharyngeal and nonpharyngeal GAS infections. Isolates were recovered in 596 noninvasive disease cases from cultures of throat swab (n = 541, 89.6%), skin and soft tissue exudates (pus) (n = 30, 5.0%) or purulent middle ear fluid (n = 25, 4.1%), and in eight cases of invasive disease, from blood (n = 7, 1.1%) or synovial fluid (septic arthritis: n = 1, 0.2%) culture. The diagnoses of seven bacteremic children were meningitis (n = 1), pneumonia with empyema (n = 1), septic arthritis (n = 1), osteomyelitis (n = 1), and bacteremia without an identified focus of infection (n = 3). Children were aged 0.8–15.5 years (median 6.5 years; interquartile range 4.5–9.0 years); 57.9% were boys.

### *emm* types, subtypes and clusters of GAS isolates

Of the total 604 pharyngeal and nonpharyngeal GAS isolates obtained, 517 were viable and underwent *emm* typing; they belonged to 20 distinct *emm* types (Table 1). We were able to analyze all eight isolates that were recovered from children with invasive GAS disease in the 7-year study period.

**Table 1:** *emm* types and clusters of group A streptococcal isolates according to clinical diagnosis for the 2011–2017 period.

*emm* Cluster	*emm* Type(s)	No. of isolates^a^	Pharyngitis^a^	SSTI^a^	AOM^a^	Invasive disease^a^
		n = 517^b^	n = 465	n = 24	n = 20	n = 8
E4	89	71 (13.7)	64 (13.8)	4 (16.7)	2 (10)	1 (12.5)
	28	58 (11.2)	51 (10.9)	3 (12.5)	4 (20)	
	77	23 (4.5)	22 (4.7)		1 (5)	
	2	14 (2.7)	12 (2.6)		2 (10)	
	8	1 (0.2)	1 (0.2)			
	Total	167 (32.3)	150 (32.3)	7 (29.2)	9 (45)	1 (12.5)
A-C3	1	92 (17.8)	74 (15.9)	5 (20.8)	9 (45)	4 (50)
E1	4	65 (12.6)	62 (13.3)	3 (12.5)		
A-C4	12	60 (11.6)	56 (12.1)	2 (8.3)	1 (5)	1 (12.5)
A-C5	3	56 (10.8)	56 (12.1)			
E6	75	30 (5.8)	28 (6)	1 (4.2)		1 (12.5)
	11	11 (2.1)	10 (2.2)	1 (4.2)		
	48	2 (0.4)	1 (0.2)	1 (4.2)		
	65	1 (0.2)		1 (4.2)		
	Total	44 (8.5)	39 (8.4)	4 (16.7)		1 (12.5)
M6	6	24 (4.6)	21 (4.5)	2 (8.3)		1 (12.5)
E3	87	4 (0.8)	3 (0.6)	1 (4.2)		
	44	1 (0.2)	1 (0.2)			
	58	1 (0.2)			1 (5)	
	118	1 (0.2)	1 (0.2)			
	Total	7 (1.4)	5 (1)	1 (4.2)	1 (5)	
E2	76	1 (0.2)	1 (0.2)			
D4	192	1 (0.2)	1 (0.2)			

^a^ Number, percent in parentheses

^b^ 517 *emm* typed isolates out of 604 recovered

SSTI: Skin and soft tissue infection

AOM: Acute otitis media

The seven most prominent *emm* types among pharyngeal isolates were *emm*1, *emm*89, *emm*4, *emm*12, *emm*3, *emm*28 and *emm*75, which accounted for 84.1% of the isolates. Among nonpharyngeal isolates, the most prominent types were *emm*1, *emm*89, *emm*28, *emm*12, *emm*4, *emm*6, *emm*2 and *emm*75, which accounted for 88.5% of isolates in this group.

The 71 *emm*89 isolates (Table 1) were negative for the presence of the *hasABC* locus and are therefore presumed to belong to the recently emerged acapsular clade [33].

In addition to subtype *emm*.0, the following 23 *emm* subtypes were detected: *emm*1.16 (n = 2), *emm*1.24 (n = 1), *emm*1.119 (n = 1), *emm*1.120 (n = 2), *emm*3.1(n = 45), *emm*3.24 (n = 1), *emm*3.77 (n = 9), *emm*3.150 (n = 1), *emm*4.19 (n = 4), *emm*6.4 (n = 15), *emm*6.54 (n = 8), *emm*6.102 (n = 1), *emm*11.1(n = 1), *emm*12.1 (n = 1), *emm*12.19 (n = 2), *emm*12.37 (n = 4), *emm*12.4 (n = 5), *emm*12.102 (n = 2), *emm*28.21 (n = 6), *emm*48.1 (n = 2), *emm*58.7 (n = 1), *emm*65.6 (n = 1) and *emm*118.12 (n = 1).

Analysis of *emm* types according to *emm* cluster classification indicated that 20 *emm* types belonged to 10 *emm* clusters (Table 1). The most prevalent *emm* clusters were E4, A-C3, E1, A-C4 and A-C5, which, in total, accounted for 85.1% of all (pharyngeal and nonpharyngeal) isolates.

### Yearly fluctuation of *emm* types and clusters

There was yearly fluctuation in the predominance of *emm* types. The variation in those belonging to *emm*4, *emm*89, *emm*12 and *emm*28 reached statistical significance (*P*<0.05).

**Fig 1:**
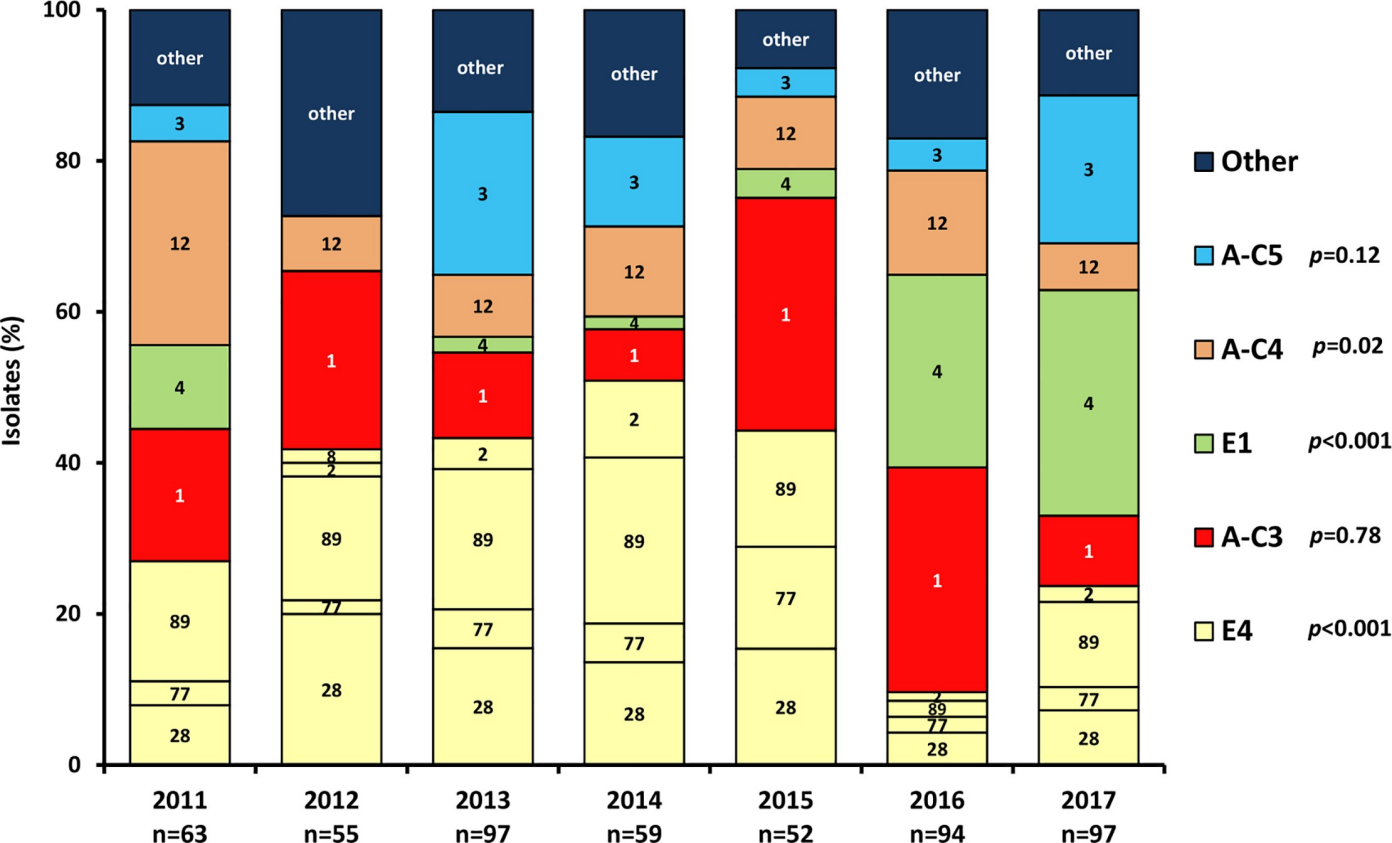
Annual frequencies of group A streptococcal isolates according to *emm* cluster and *emm* type in the 517 cases studied. Only the five most common (>10%) *emm* clusters are represented with the *P* values of their fluctuation, while the five least common ones are grouped as ‘Other’. Numbers inside bars represent *emm* types in each cluster. Bar colors are explained in the Figure.

Yearly variation of the *emm* clusters and level of significance in the fluctuation of pharyngeal and nonpharyngeal isolates within these clusters is presented in Fig 1. Significant yearly variation was also observed in clusters E4 (*P*<0.001), E1 (*P*<0.001) and A-C4 (*P* = 0.028) among pharyngeal isolates and in cluster E1 (*P* = 0.035) among nonpharyngeal noninvasive ones.

### Macrolide resistance and MLST typing

**Fig 2:**
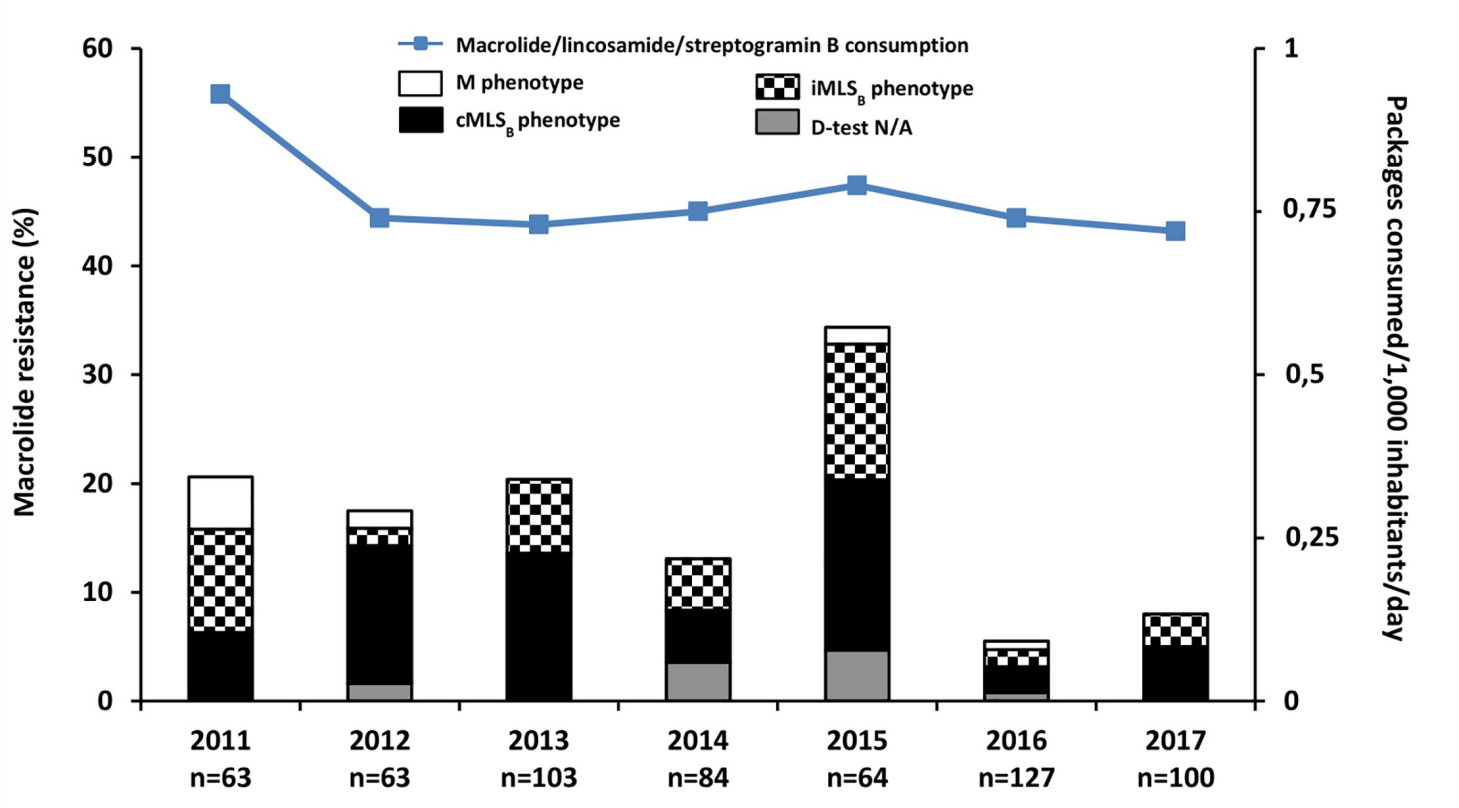
Annual macrolide resistance rates and resistance phenotypes among 604 group A streptococcal isolates in Central Greece (2011–2017). The continuous line indicates the national outpatient consumption of total macrolide, lincosamide and streptogramin B packages (tablets and suspensions); IMS data are expressed as packages consumed per 1,000 inhabitants per day. Data on national outpatient consumption in the period preceding the current survey (2007–2010) are given in the text. The resistance phenotype is not available in eight isolates, as follows: 2012 (n = 1), 2014 (n = 3), 2015 (n = 3), and 2016 (n = 1).

All GAS isolates were susceptible to benzylpenicillin. The number of GAS isolates recovered each year, the frequency of macrolide resistance and the resistance phenotypes appear in Fig 2. Over the 7-year study period, resistance to macrolides was found in 93 (15.4%) of the collected 604 GAS isolates. The great majority of these macrolide-resistant isolates (92.9%) exhibited either the iMLS_B_ or the cMLS_B_ phenotype. Specifically, 56.4% had the cMLS_B_ phenotype, 36.5% the iMLS_B_ and 7.1% the M phenotype (Fig 2). There was no significant difference in macrolide resistance between pharyngeal isolates and those from other noninvasive infections (15.5% versus 16.4%, *P* = 0.85). All eight isolates recovered from children with invasive disease (n = 8) were macrolide susceptible.

During the study period, we noted a tendency towards significant decline in resistance rate (χ^2^ for trend *P* = 0.002). The lowest rates occurred in years 2014 (13.1%), 2016 (5.5%) and 2017 (8.0%). However, in 2015, an increased circulation of the cMLS_B_ and iMLS_B_ phenotype isolates was noted (Fig 2).

Among the 517 *emm* typed isolates, 85 were macrolide-resistant and were genotyped. They belonged to a limited number of *emm* types (Table 2); *emm*28, *emm*77 and *emm*12 accounted for 81.2% of the macrolide-resistant GAS isolates. Specifically, *emm*77 and *emm*4 accounted for 90.3% of the isolates carrying *erm*(TR), either alone or in combination with *mef*(A); *emm*28 and *emm*12 accounted for 87.5% of those carrying *erm*(B), either alone or in combination with *mef*(A) (Table 2).

**Table 2:** *emm* types, resistance genotypes and STs of 85 macrolide-resistant group A streptococcal isolates.

*emm* Type	No. of isolates	STs^a^^,^^b^	Macrolide resistance genotype				
			*erm*(B)^c^	*erm*(B)plus *mef*(A)^c^	*erm*(TR)^c^	*erm*(TR)plus *mef*(A)^c^	*mef*(A)^c^
emm28	37	ST52 [36]	29 (80.6)	7 (19.4)			
		ST1117 [1]^d^					1 (100)
*emm*77	23	ST63			21 (91.3)	2 (8.7)	
*emm*12	9	ST36	5 (55.6)	1 (11.1)	1 (11.1)	1 (11.1)	1(11.1)
*emm*89	5	ST101	1 (20)		1 (20)		3 (60)
*emm*4	5	ST39			5 (100)		
*emm*11	3	ST20	3 (100)				
*emm*1	1	ST28					1 (100)
*emm*75	1	ST150		1 (100)			
*emm*58	1	ST176	1 (100)				
Total	85	85	39 (45.9)	9 (10.6)	28 (32.9)	3 (3.5)	6 (7.1)

^a^ STs: Sequence Types

^b^ In brackets: Number of isolates

^c^ Number, percent in parentheses

^d^ Novel ST

In 2015, we noted an increase of the four most prominent types of macrolide-resistant isolates (*emm*77, *emm*28, *emm*12 and *emm*89).

All macrolide-resistant isolates that possessed *erm*(B), alone or in combination with *mef*(A), exhibited the cMLS_B_ phenotype. All those possessing *erm*(TR), alone or in combination with *mef*(A), exhibited the iMLS_B_ phenotype. Of those harboring solely *mef*(A) all exhibited the M phenotype.

Macrolide-resistant isolates were studied by MLST (Table 2). One *emm*28 isolate with a new allele combination, which produced a novel ST −ST1117−, was detected in a pharyngeal sample. In this new ST1117, the *gki* locus was 99% identical to allele 11. The difference was a T to C alteration at position 458bp and was assigned in MLST database as 172 allele number. All other isolates of each *emm* type tested belonged to a single ST.

Of the 71 *emm*89 isolates, 66 (93%) were macrolide susceptible. All seven typed ‘new clade’ macrolide-susceptible isolates were ST101, as follows: one from blood culture (bacteremia without an identified focus of infection), two from skin and soft tissue exudate, one from middle ear exudate and three pharyngeal swab specimens.

### Associations between *emm* types and macrolide resistance

Of the 517 *emm* typed isolates, those that were macrolide-susceptible belonged to 18 *emm* types and the macrolide-resistant ones to nine *emm* types. Statistical associations between *emm* types and macrolide resistance are depicted in Table 3.

**Table 3:** *emm* type distribution of the macrolide-susceptible and macrolide-resistant group A streptococcal isolates.

*emm* Type	n	Macrolide-susceptible^a^	Macrolide-resistant^a^	Residual^b^	*P* value	Adjusted *P* value^c^
*emm*1	92	91 (21.1)	1 (1.2)	-4.4	**<0.001**	**<0.001**
*emm*89	71	66 (15.3)	5 (5.9)	-2.3	**0.021**	0.300
*emm*4	65	60 (13.9)	5 (5.9)	-2.0	**0.049**	0.586
*emm1*2	60	51 (11.8)	9 (10.6)	-0.3	0.749	1.000
*emm*28	58	21 (4.9)	37 (43.5)	10.3	**<0.001**	**<0.001**
*emm*3	56	56 (13.0)	0 (0)	-3.5	**<0.001**	0.006
*emm7*5	30	29 (6.7)	1 (1.2)	-2.0	**0.046**	0.644
*emm*6	24	24 (5.5)	0 (0)	-2.2	**0.026**	0.365
*emm*77	23	0 (0)	23 (27)	11.1	**<0.001**	**<0.001**
*emm*2	14	14 (3.2)	0 (0)	-1.7	0.092	1.000
*emm*11	11	8 (1.8)	3 (3.5)	1.0	0.327	1.000
*emm*87	4	4 (0.9)	0 (0)	-0.9	0.373	1.000
*emm*48	2	2 (0.5)	0 (0)	-0.6	0.530	1.000
Other	7	6 (1.4)^d^	1 (1.2)^e^	-0.2	0.877	1.000
Total	517	432 (100.0)	85 (100.0)			

^a^ Number, percent in parentheses

^b^ Standardized residuals method [36] for the significance of difference between macrolide-susceptible and macrolide-resistant rates

^c^ Bonferroni correction for multiple comparisons

^d^ *emm* Type (number of isolates): *emm*8 (n = 1); *emm*44 (n = 1); *emm*65 (n = 1); *emm*76 (n = 1); *emm*118 (n = 1); *emm*192 (n = 1)

^e^ *emm* Type (number of isolates): *emm*58 (n = 1)

### Consumption of macrolides

In addition to the data obtained during the study period on outpatient macrolide, lincosamide and streptogramin B consumption (Fig 2), we also obtained information on their use in previous years. At a national level, a substantial decrease (26.6%) in macrolide, lincosamide and streptogramin B consumption was noted during 2007–2010, from 1.24 packages/1,000 inhabitants/day in 2007 to 0.91 packages/1,000 inhabitants/day in 2010 (authors’ unpublished data based on IMS). The decline in consumption between 2011 and 2017 was 22.6%; the greatest decline (20.4%) was due to the decrease during the 2011–2012 period (Fig 2).

Specifically, the total macrolide, lincosamide and streptogramin B package consumption (tablets and suspensions) in Greece showed a reduction from 3,761,780 in 2011 to 2,835,653 packages in 2017 (24.6% decrease, IMS Health data). During the same time period, consumption of pediatric suspension packages also decreased from 840,981 packages in 2011 to 541,893 packages in 2017 (35.6% decrease, IMS Health data). During the 7-year study period, there was no association between annual macrolide resistance and macrolide, lincosamide and streptogramin B consumption regardless of whether use of both tablets and suspensions (Pearson’s correlation r = 0.404, *P* = 0.369) or suspensions alone (Pearson’s correlation r = 0.473, *P* = 0.283) were taken into account.

### *emm* types of the isolates in relation to an experimental 30-valent GAS vaccine

**Fig 3:**
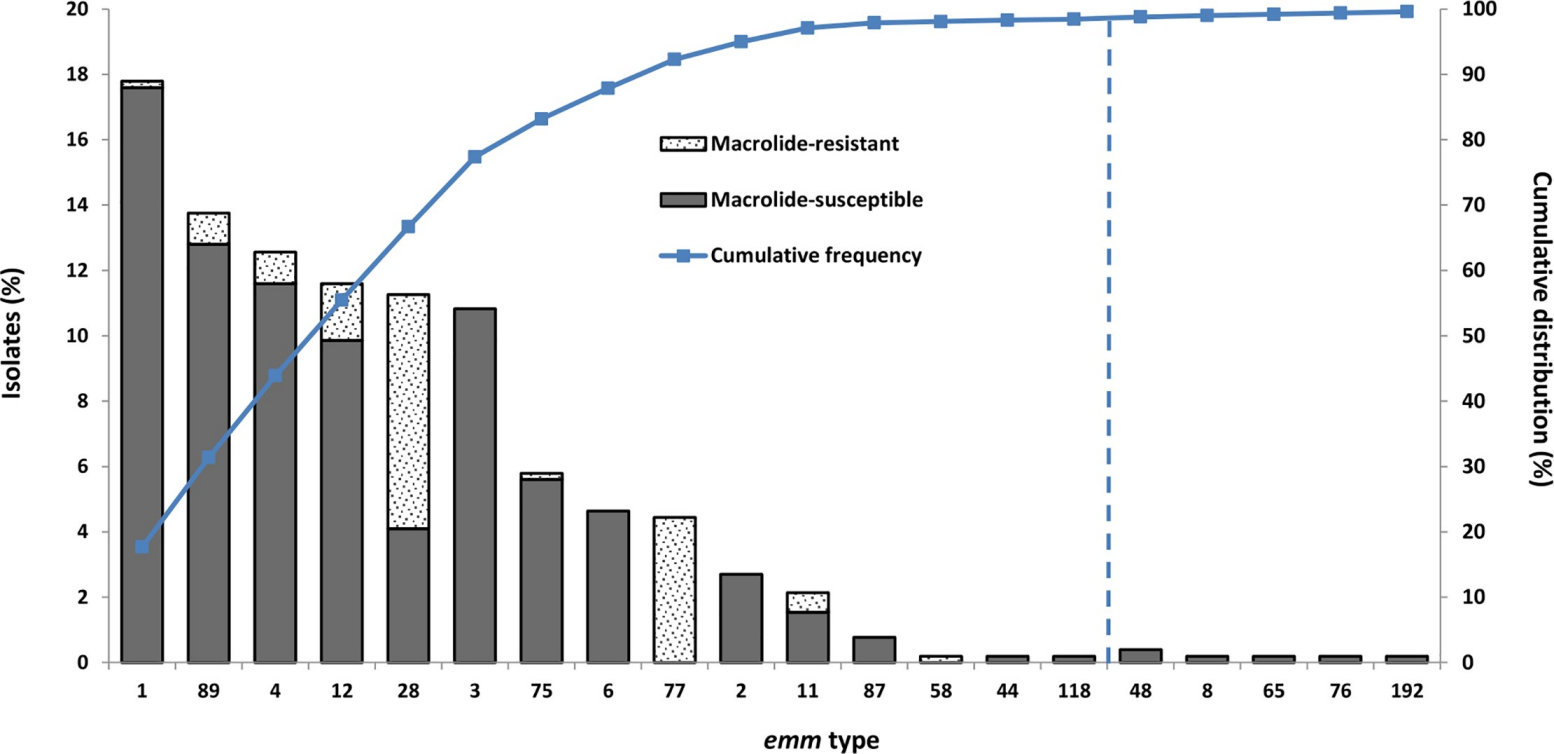
Cumulative distribution of *emm* types among 517 group A streptococcal isolates during 2011–2017 and its relation to the 30-valent vaccine.

Fifteen of the 20 *emm* types found in our population −both pharyngeal and nonpharyngeal− are included in the 30-valent M protein vaccine under development. The estimated overall coverage rate by this vaccine is 98.8% of the isolates identified in our study. The cumulative distribution of *emm* types of GAS isolates is presented in Fig 3. Four hundred and sixty-one (99.1%) of the 465 pharyngeal and 50 (96.2%) of the 52 nonpharyngeal isolates belonged to *emm* types that are included in the proposed vaccine.

## Discussion

We analyzed 604 GAS isolates recovered from pediatric patients in the area of Central Greece from 2011 through 2017. Twenty distinct *emm* types comprised 10 *emm* clusters, among which −in descending order− E4, A-C3, E1, A-C4 and A-C5 prevailed. We found that the macrolide resistance rate was 15.4% in this 7-year period. The cMLS_B_ phenotype predominated and persisted throughout the study, whereas the prevalence of M phenotype was 8-fold lower. The present survey updates available information on GAS in this area of Central Greece obtained by the same laboratory in two previous surveys, in 2001 [37] and in 2007–2009 [38]. Our recent findings show lower macrolide resistance rates than those found in the two previous surveys and an inversion of the dominant resistance phenotypes (S1 Table). The resistance rate increased from 19.3% in 2001 [37] to 24% in 2007–2009 [38] and dropped in the recent 2011–2017 survey. Moreover, the macrolide resistance rates in the two most recent years of the present study remained below 10%. Nevertheless, it should not be assumed that Greece has entered a sustained decline of macrolide resistance, and surveillance of GAS macrolide resistance should be maintained in the years to follow before safe conclusions can be drawn.

Among pharyngeal isolates, the seven most frequent *emm* clusters accounted for 98.5% of the *emm* typed isolates in this study. Other reports have shown similar results: 96.5% of those collected in a prospective study conducted in Western Greece between 1999 and 2005 [29], authors’ unpublished data], 92.8% of 7,040 isolates in the USA [12] and 91.1% in Southern Taiwan [39]. High diversity of *emm* types has been observed in the Pacific region and other low-income settings [11, 40].

A clade of *emm*89 isolates lacking the hyaluronic acid capsule biosynthesis locus has emerged in the 2000s and spread throughout several countries in North America and Europe [33, 41]. Seventy-one *emm*89 isolates were tested and were all inferred to belong to the new clade based on the absence of capsule locus. Seven percent of these was macrolide resistant and exhibited various phenotypes and resistance genotypes. The 12 tested isolates −seven macrolide-susceptible and five macrolide-resistant− belonged to ST101, which is the most common type among the acapsular clade [41]. Given the high prevalence of *emm*89 in this study, it appears that this clade has also become dominant in central Greece and macrolide resistance has emerged in selected isolates.

A significant association was found between macrolide susceptibility and *emm*1. We also established significant associations between macrolide resistance and the *emm*28 and *emm*77 types. Other studies report a similar correlation for *emm*28 [25–27, 30, 41] and *emm*77 [25, 28, 30, 42].

The distribution of our most common macrolide-resistant clones, −*emm*28/ST52, *emm*77/ST63, *emm*12/ST36, emm89/ST101 and *emm*4/ST39− varied widely in different European regions over time, while certain macrolide-resistant lineages frequently encountered in some European countries −*emm*22/ST46, *emm*11/ST403 and *emm*1/ST28− were either absent or uncommon in our study [25–28, 43–46]. Of note, in our survey, macrolide-resistant *emm*4/ST39 GAS lineage exhibited iMLS_B_ phenotype, whereas in other European countries, this lineage has been most frequently found among resistant isolates which expressed the M phenotype [25, 26, 43–45].

Temporal changes in clonal composition have been noted in Central Greece. In the previous survey conducted in the same region between January 2007 and June 2009, no isolates harboring *erm*(B) gene and expressing cMLS_B_ phenotype occurred; *emm*28/ST52 and *emm*58/ST176 were absent, whereas *emm*89/ST101, emm12/ST36 and *emm*75/ST150 were identified among macrolide-susceptible isolates [38]. The lineages *emm*94/ST89 and *emm*77/ST550 identified in the previous survey were not detected in this recent one (S1 Table). High clonal instability has been reported in Portugal from 2007 to 2013 [27].

Greece has been, and remains, an outstanding macrolide consumer in Europe [47]. At a national level, a gradual decline in the outpatient macrolide, lincosamide and streptogramin B consumption was noted between 2007 and 2010 and was further enhanced in 2012. From 2013 onwards, there has been limited change. A 0.67 value of packages/1,000 inhabitants/day of macrolide, lincosamide, streptogramin B and tetracycline has been proposed as representing a critical threshold. It was shown that when the use of these classes of antimicrobial agents was maintained below this critical threshold, a low level of macrolide-resistant GAS isolates and an increased number of *erm*(A)-harboring *emm*77 GAS with low fitness costs occurred [35]. The national consumption has not been lowered to this threshold in Greece. This may provide an explanation for the dominance of the *erm*(B)-associated cMLSB phenotype in Greece despite the reduction in the macrolide resistance rate. The small (2%) difference in macrolide, lincosamide and streptogramin B consumption between the two calculation methods, i.e., crude number of packages *versus* packages/1,000 inhabitants/day index, is due to small population changes during the study years and the logistics of the calculations.

In the surveillance period of our study the lowest rates of macrolide resistance were observed in 2014 (13.1%), 2016 (5.5%) and 2017 (8.0%). This decrease may be attributed to the gradual reduction in the consumption of macrolides, which commenced in the pre-study period, i.e. 2007–2010, in association with the dynamics of specific major macrolide-resistant clones [26, 27]. We speculate that the niche of macrolide-resistant clones that remains in a community after such reduction may occasionally lead to a temporary increased spread of these clones and cause a transient surge of macrolide resistance rate, similar to the one observed in our study during 2015. The subsequent nadir rates observed in 2016 and 2017 are echoed by the results of other independent studies including an abrupt drop from a high rate of approximately 22% in 2012 to a low one of 10.95% in 2013 reported by a major Children’s Hospital in Athens [30], and a similar reduction from a high rate of 16.3% in 2008 to a low one of 5.1% in 2009 in a region of Central Italy [28].

Finally, according to our study data, a 98.8% coverage of the isolates would be achieved by the 30-valent GAS vaccine under development. A 93.7% coverage by the same vaccine has been estimated for northern Spain [48]. It is evident that the potential efficacy of vaccines based on the M protein is limited by their suitability to local epidemiological characteristics.

There are certain limitations to this study. First, it is a single-center study. However, our tertiary care Center serves a substantial proportion of the pediatric population of Central Greece. Second, in our analysis we compared the regional macrolide resistance rate with the total national macrolide consumption, as it was not feasible to obtain an accurate estimation of the consumption −of pediatric packaging (suspension), in particular− at the local level. On the other hand, there are important strengths to our study. It is comparable to two previous surveys and all three studies span a period of 17 years (2001–2017). Indeed, these surveys have shared the same region of surveillance and method of sampling, and all samples have been analyzed by the same laboratory.

In conclusion, in Central Greece during 2011–2017, macrolide resistance rate of GAS isolates decreased to 15.4% as compared to 19.3% in 2001 and 24% in 2007–2009; it remained below 10% in the last two years of the study. During the study period the cMLS_B_ phenotype predominated. A relatively limited number of *emm* types prevailed among GAS isolates recovered from children with pharyngeal and nonpharyngeal infections. A yearly fluctuation in the frequency of *emm* types and clusters was evident. The most frequently found genetic lineages of macrolide-resistant GAS isolates include those defined by *emm*28/ST52, *emm*77/ST63, *emm*12/ST36, *emm*89/ST101 and *emm*4/ST39. Interestingly, 98.8% of the GAS isolates recovered in our population belonged to *emm* types that are included in the 30-valent M protein vaccine under development. We propose that molecular surveillance of GAS isolates at different time-periods and in various areas, especially in the event of increased prevalence of macrolide-resistant GAS, may guide treatment strategies and the formulation and targeting of multivalent type-specific vaccines.

## Supporting information

S1 Table Comparison of GAS isolates recovered in Central Greece in 2001, 2007–2009 and 2011–2017. (PDF)
