# Supplementary material for: emm Types and clusters and macrolide resistance of pediatric group A streptococcal isolates in Central Greece during 2011-2017
Source: PLoS One. 2020 May 7;15(5):e0232777. doi: 10.1371/journal.pone.0232777 (PMC7205280; doi:10.1371/journal.pone.0232777)
Supplement: S1 Table — (PDF) [file pone.0232777.s001.pdf]

Supplementary Table 1: Comparison of GAS isolates recovered in Central Greece in 2001, 2007-2009 and 2011-2017.

	Petinaki et al. [36]	Malli et al. [37]	Current study
Study period	1/2001 - 12/2001	1/2007 - 6/2009	1/2011 - 12/2017
Region	Central Greece	Central Greece	Central Greece
Number of macrolide-resistant/total isolates[a]	58/300 (19.3)	119/495 (24)	93/604 (15.4)
Resistance phenotype available	58	119	85
M	40 (68.9)	20 (17.0)	6 (7.1)
iMLS$_B$	12 (20.7)	99 (83.0)	31 (36.5)
cMLS$_B$	6 (10.3)	0	48 (56.4)
Resistance determinants available	58	119	85
mef(A)	40 (69.0)	20 (17.0)	6 (7.0)
erm(TR)	14 (24.1)	99 (83.0)	28 (33.0)
erm(TR) & *mef*(A)	0 (0)	0 (0)	3 (3.5)
erm(B)	4 (7.0)	0 (0)	39 (45.9)
erm(B) & *mef*(A)	0 (0)	0 (0)	9 (10.6)
Number of macrolide-susceptible (MS) and macrolide-resistant (MR) *emm* typed isolates	N/A[b]	MS (n=120), MR (n=119)	MS (n=432), MR (n=85)
Four most frequent *emm* types among MS isolates by descending order		*emm*89, *emm*1, *emm*3, *emm*4	*emm*1, *emm*89, *emm4*, *emm*3
Four most frequent *emm* types among MR isolates by descending order		*emm*4, *emm*77, *emm*94[c], *emm*1[c]	*emm*28, *emm*77, *emm*12, *emm*89[c], *emm*4[c]
Clonal analysis of MR isolates by *emm* typing and MLST[d]	N/A	119	85
MR clones by descending order		*emm*4/ST39, *emm*77/ST63, *emm*1/ST28[c], *emm*94/ST89[c], *emm*77/ST550[c]	*emm*28/ST52, *emm*77/ST63, *emm*12/ST36, *emm4*/ST39[c], *emm*89/ST101[c], *emm*11/ST20, *emm*1/ST28[c], *emm*75/ST150[c], *emm*58/ST176[c], *emm*28/ST1117[c]

[a] Numbers in parentheses are percent unless otherwise indicated; [b] N/A: Not available; [c] Equal number of isolates ; [d] MLST: Multilocus Sequence Typing
